# Effect of a multimodal strategy for prevention of nosocomial influenza: a retrospective study at Grenoble Alpes University Hospital from 2014 to 2019

**DOI:** 10.1186/s13756-021-01046-y

**Published:** 2022-02-08

**Authors:** Meghann Gallouche, Hugo Terrisse, Sylvie Larrat, Sylvie Marfaing, Christelle Di Cioccio, Bruno Verit, Patrice Morand, Vincent Bonneterre, Jean-Luc Bosson, Caroline Landelle

**Affiliations:** 1 MESP TIM-C UMR 5525, Univ. Grenoble Alpes/CNRS, Grenoble INP, Grenoble, France; 2 Service d’hygiène hospitalière, CHU Grenoble Alpes, Grenoble, France; 3 Laboratoire de virologie, CHU Grenoble Alpes, Grenoble, France; 4 Direction des soins, CHU Grenoble Alpes, Grenoble, France; 5 Service de santé au travail, CHU Grenoble Alpes, Grenoble, France; 6 Institut de biologie structurale, UMR 5075, Univ. Grenoble Alpes/CNRS/CEA, Grenoble, France; 7 EPSP TIM-C UMR 5525, Univ. Grenoble Alpes/CNRS, Grenoble INP, Grenoble, France; 8 Pôle de Santé Publique, CHU Grenoble Alpes, Grenoble, France

**Keywords:** Healthcare-associated infection, Nosocomial influenza, Infection control, Multimodal strategy, Prevention

## Abstract

**Background:**

A multimodal strategy to prevent nosocomial influenza was implemented in 2015–2016 in Grenoble Alpes University Hospital. Three modalities were implemented in all units: promotion of vaccination among healthcare workers, epidemiologic surveillance and communication campaigns. Units receiving a high number of patients with influenza implemented 2 additional modalities: improvement of diagnosis capacities and systematic surgical mask use. The main objective was to assess the effectiveness of the strategy for reducing the risk of nosocomial influenza.

**Methods:**

A study was conducted retrospectively investigating 5 epidemic seasons (2014–2015 to 2018–2019) including all patients hospitalized with a positive influenza test at Grenoble Alpes University Hospital. The weekly number of nosocomial influenza cases was analyzed by Poisson regression and incidence rate ratios (IRR) were estimated.

**Results:**

A total of 1540 patients, resulting in 1559 stays, were included. There was no significant difference between the 5 influenza epidemic seasons in the units implementing only 3 measures. In the units implementing the 5 measures, there was a reduction of nosocomial influenza over the seasons when the strategy was implemented compared to the 2014–2015 epidemic season (IRR = 0.56, 95% CI = 0.23–1.34 in 2015–2016; IRR = 0.39, 95% CI = 0.19–0.81 in 2016–2017; IRR = 0.50, 95% CI = 0.24–1.03 in 2017–2018; IRR = 0.48, 95% CI = 0.23–0.97 in 2018–2019).

**Conclusions:**

Our data mainly suggested that the application of the strategy with 5 modalities, including systematic surgical mask use and rapid diagnosis, seemed to reduce by half the risk of nosocomial influenza. Further data, including medico-economic studies, are necessary to determine the opportunity of extending these measures at a larger scale.

## Background

Each year, 5 to 10% of the world’s population are affected with seasonal influenza resulting in 290,000 to 650,000 deaths [1]. In France, on average, 2.5 million persons are infected each year with several thousands of deaths directly attributable to influenza [2].

The influx of patients generated by seasonal influenza can exceed the capacities of care in hospitals, as it happened at Grenoble Alpes University Hospital in 2014–2015. Moreover, acquisition of influenza within healthcare facilities is a concern given the high proportion of patients at risk of severe influenza [3]. Nosocomial influenza outbreaks have been described in literature [4–6] and the role of healthcare workers (HCWs) in these outbreaks has been suggested [7, 8].

Although evidence is limited on vaccination providing a direct protection against influenza for HCWs [9], there might be benefits for the patients with an indirect protection through HCWs’ vaccination [10]. It could also reduce the risk of nosocomial influenza [11, 12]. HCWs’ vaccination is thus generally recommended [13, 14], but it does not appear sufficient to prevent influenza transmission and should rather be part of a larger strategy [15]. Among other potential measures, surgical mask use could prevent the transmission of respiratory pathogens. However, its effectiveness remains unclear and acceptability may be a concern [16, 17]. The use of rapid molecular assays for diagnosis also appears as an important tool to manage influenza outbreaks [18–21]. As stated in guidelines [22, 23], when patients are hospitalized with influenza, control measures (mainly single occupancy room and surgical mask for HCWs) must be taken to prevent droplet transmission. Faster the diagnosis is made, faster these precautions can be implemented, thereby reducing the risk of transmission. Guidelines for the management of seasonal influenza in healthcare settings also recommend implementing an active surveillance to detect any increased influenza activity and to apply control measures if necessary [24, 25].

Review of literature provides many reports on the impact of one or two preventive measures. However, the benefit of multimodal strategies has been previously suggested for preventing healthcare-associated infections [26, 27]. A multimodal strategy to prevent nosocomial influenza was thus implemented from the 2015–2016 epidemic season. Three measures were implemented in all units: promotion of HCWs’ vaccination, epidemiologic surveillance and communication. Units receiving many patients with influenza implemented 2 additional measures: improvement of diagnosis capacities and systematic surgical mask use for HCWs and visitors.

The main objective was to assess the impact of a 3 to 5-steps multimodal prevention strategy on reducing the risk of nosocomial influenza in a University Hospital over 5 epidemic seasons from 2014 to 2019. Secondary objectives were to evaluate the impact of the multimodal strategy on HCWs’ vaccination rates and to assess conformity of masks use in designated units.

## Method

### Patients and settings

Grenoble Alpes University Hospital is a 2133-bed French hospital with over 110,000 emergency stays and 550,000 inpatient days in 2018. A retrospective study was conducted including all patients over 18 years old, who had a positive influenza test at Grenoble Alpes University Hospital and who were hospitalized between November 2014 and April 2019, during an epidemic period. If a patient had several hospital stays corresponding to different influenza episodes, each hospitalization was analyzed. Pediatric cases, cases occurring outside of influenza epidemic periods and patients who were not fully hospitalized (emergency stays only, consultations, ambulatory surgery etc.) were excluded. An epidemic period was defined as ≥ 10 cases per week within the hospital. Five epidemic seasons were considered: 2014–2015 (when full epidemic surveillance was not implemented yet), 2015–2016, 2016–2017, 2017–2018 and 2018–2019.

### Description of the strategy

A 3 to 5-steps multimodal strategy to prevent nosocomial influenza was implemented from the 2015–2016 epidemic season. Three measures were implemented in all units (n = 78): promotion of HCWs’ vaccination, epidemiologic surveillance and communication. Two additional measures, improvement of diagnosis capacities and systematic surgical mask use for HCWs and visitors, were implemented in units which were more susceptible to receive patients with influenza (risk units) only. Risk units included adult emergency department, geriatrics, internal medicine, infectious diseases, post-emergency (multidisciplinary medical unit for patients admitted through the emergency department), and pathological pregnancies (n = 15). Further details on the strategy are provided in Table 1. The choice of surgical masks was based on national recommendations given the absence of superiority of FFP2 respirator masks and a greater tolerance with surgical masks [22]. The use of FFP2 respirator masks was restricted to aerosol-generating procedures [22].

**Table 1:** Description of the 3 to 5-steps multimodal strategy for nosocomial influenza prevention

Measure	Details and evolution	Units	Evaluation
Promotion of vaccination among HCWs	- Communication and information (emails, posters etc.) on vaccination	All units	Vaccination rate among HCWs determined with data from the occupational health unit
	- Vaccination in the occupational health unit during all epidemic seasons		
	- Delocalized vaccination in units by a referent nurse was implemented from 2017–2018 onwards		
	- A survey on vaccine hesitation was carried out during the 2017–2018 epidemic season		
	- Delocalized vaccination at the hospital lunchroom was implemented from 2018–2019 onwards		
	- Interventional studies with implementation intention or impact of threat were carried out during the 2018–2019 and 2019–2020 epidemic seasons [31]		
Implementation of an epidemiologic surveillance	- Daily surveillance of the number of cases within the hospital	All units	Conformity rate for adherence to droplet precautions
	- Collection of information for all patients with a positive influenza test		
	- Check of droplet precautions’ application and oseltamivir treatment and reminder if necessary		
	- Determination of nosocomial status by an infection control practitioner		
	- Outbreak control measures if > 2 cases with nosocomial transmission within one unit		
Communication	- Local recommendation available about management of influenza cases (precautions, treatment, outbreak management, etc.…)	All units	NA
	- Communication on influenza and vaccination to HCWs, patients and visitors with posters dispatched within the hospital		
	- Weekly emails on vaccination for HCWs		
	- Feed-back: weekly feedback to staff on outbreak evolution through the intranet portal during the epidemic period, reports in institutional commissions and in risk units at the end of the epidemic		
Implementation of systematic surgical masks use	- Systematic surgical masks use	Risk units*	Conformity rate for HCWs and visitors determined with biweekly audits in risk units during the epidemic period
	- Implementation for all HCWs and visitors regardless of their vaccination status for the duration of the epidemic period		
	- Benchmarking (weekly feedback for each unit with comparison to the global results)		
Improvement of diagnosis capacities	- Serial tests with RT-PCR R-DiaFlu^®^ (≈ 5 h, performed in the virology laboratory on weekdays and Saturday mornings): used during the 5 epidemic seasons	Risk units*	Number of tests performed during the epidemic period
	- Rapid tests with RT-PCR GeneXpert^®^ (≈ 35 min, performed in the virology laboratory):		
	- for emergencies only in 2014–2015		
	- in routine practice for risk units in 2015–2016 (weekdays and Saturday mornings)		
	- in routine practice for risk units and ICUs, and on specific demand for non-risk units with justification by clinician from 2016–2017 onwards (weekdays and Saturday mornings only in 2016–2017 and 2017–2018, extended to nights and weekends in 2018–2019)		
	- Rapid point-of-care tests with Cobas^®^ Liat System (≈ 20–25 min, performed in the ED): assessment over a 2-weeks period during the 2017–2018 season [32]; then fully available during the 2018–2019 epidemic season		
	- Respifinder^®^ 2Smart (≈ 4–5 days, performed in the virology laboratory on weekdays): used during the 5 epidemic seasons used during all epidemic seasons for patients hospitalized in intensive care units (ICUs) or on specific demand		

Table presents a description of the 3 to 5-steps multimodal strategy that was implemented at Grenoble Alpes University Hospital for the prevention of nosocomial influenza from the 2015–2016 epidemic season onwards

*HCW* healthcare worker, *NA* not applicable, *ICU* intensive care unit, *ED* emergency department

*Risk units: adult emergency department, geriatric units, internal medicine units, infectious diseases unit, post-emergency unit, pathological pregnancies unit

Management measures for influenza cases included droplet precautions (single occupancy room and surgical mask use for HCWs and visitors when entering the patient’s room, if not implemented yet in the unit) and a curative treatment by Oseltamivir was prescribed if the diagnosis was made within 48 h after symptoms onset or in case of severe symptoms. When a nosocomial case was identified, contact patients were looked for and received a prophylactic treatment by Oseltamivir. If > 2 cases of nosocomial transmission occurred within one unit, measures were taken to control the cluster: continuous surgical mask use for HCWs and visitors (if not implemented yet) and prophylactic treatment for contact patients only or all patients depending on the risk of severe influenza. These measures were lifted if no new case of transmission was identified in the last 7 days. A nosocomial case was defined as a positive, influenza sample associated with symptoms onset occurring at 72 h of hospitalization or later [28].

Before the implementation of the strategy (2014–2015 season), there was no specific mask policy outside of standard hygiene precautions and droplet precautions for patients with influenza. Vaccination of HCWs was organized within the institution but the only modality was vaccination in the occupational medicine unit. A minimal surveillance was organized to identify nosocomial cases (using the same definition as later on) but without collecting further information and data were not communicated to units/HCWs. Rapid RT-PCR tests were only available for some emergencies. There was no specific communication campaign.

### Influenza diagnosis

Influenza tests were performed on patients presenting fever or feverishness and at least one of the following symptoms: headache, sore throat, cough or myalgia [29, 30]. A sample was taken using a nasopharyngeal swab sample according to the Center for Disease Control and Prevention guidelines [29, 30], and the end of the swab was placed into the liquid transport media (Sigma Virocult^®^, MWE, Wiltshire, England). Only RT-PCR tests (no antigenic tests), detecting influenza viruses A and B, were used (Table 1). In non-risk units, mainly RT-PCR with a long turnaround time were used all along the study period; rapid RT-PCR could be used for some emergencies only and if well justified by the clinician. In risk units, tests used were similar as in non-risk units for the reference season; from the 2015–2016 season onwards, rapid RT-PCR were largely available.

### Data collection

All influenza tests were retrieved from the laboratory software. Data were collected daily by the infection control unit for positive influenza tests. Collected data included age, sex, date of diagnosis and virus type for all epidemic seasons. From the 2015–2016 season onwards, collected data also included vaccination status, existence of severity risk factor(s), Oseltamivir treatment, droplet precautions prescription and the date of prescription (if not prescribed in the patient’s electronic record precautions were considered as absent), intensive care unit (ICU) stay and death. The status, nosocomial or community-acquired, was determined according to symptoms onset. Data on HCWs’ vaccination were provided by the occupational health unit. Data on surgical masks were obtained by biweekly audits carried out by the staff of infection control unit.

### Analyses

Epidemics and patients’ characteristics were described by numbers and percentages, medians and interquartile ranges (IQR) or means and standard deviations. Groups were compared by means of the Mann–Whitney test, Kruskal–Wallis test, Pearson Chi-squared test or Fisher’s exact test. Given that the number of people susceptible to be infected was not available, the proportion of nosocomial influenza among all influenza cases in hospitalization was assessed in bivariate analysis. In multivariate analysis, the weekly number of nosocomial influenza cases was analyzed by Poisson regression adjusting for total number of influenza cases, epidemic season, hospitalization in a risk unit and the interaction terms of the last two variables. Results are presented as incidence rate ratios (IRR) and their confidence interval at 95% (CI95%). Autocorrelation was assessed using an autocorrelation function plot. All tests were two-tailed and a *p* value ≤ 0.05 was considered statistically significant. Analysis was performed with Stata 12 software (StataCorp LP, College Station, TX, USA).

### Ethics

According to French policy, patient consent was not required (retrospective study) and data were declared to the Data Protection Officer of Grenoble Alpes University Hospital, France. Study ethics approval was obtained on 28/04/21 (CECIC Rhône-Alpes-Auvergne, Clermont-Ferrand, IRB 5891).

## Results

### Generalities

**Fig. 1:**
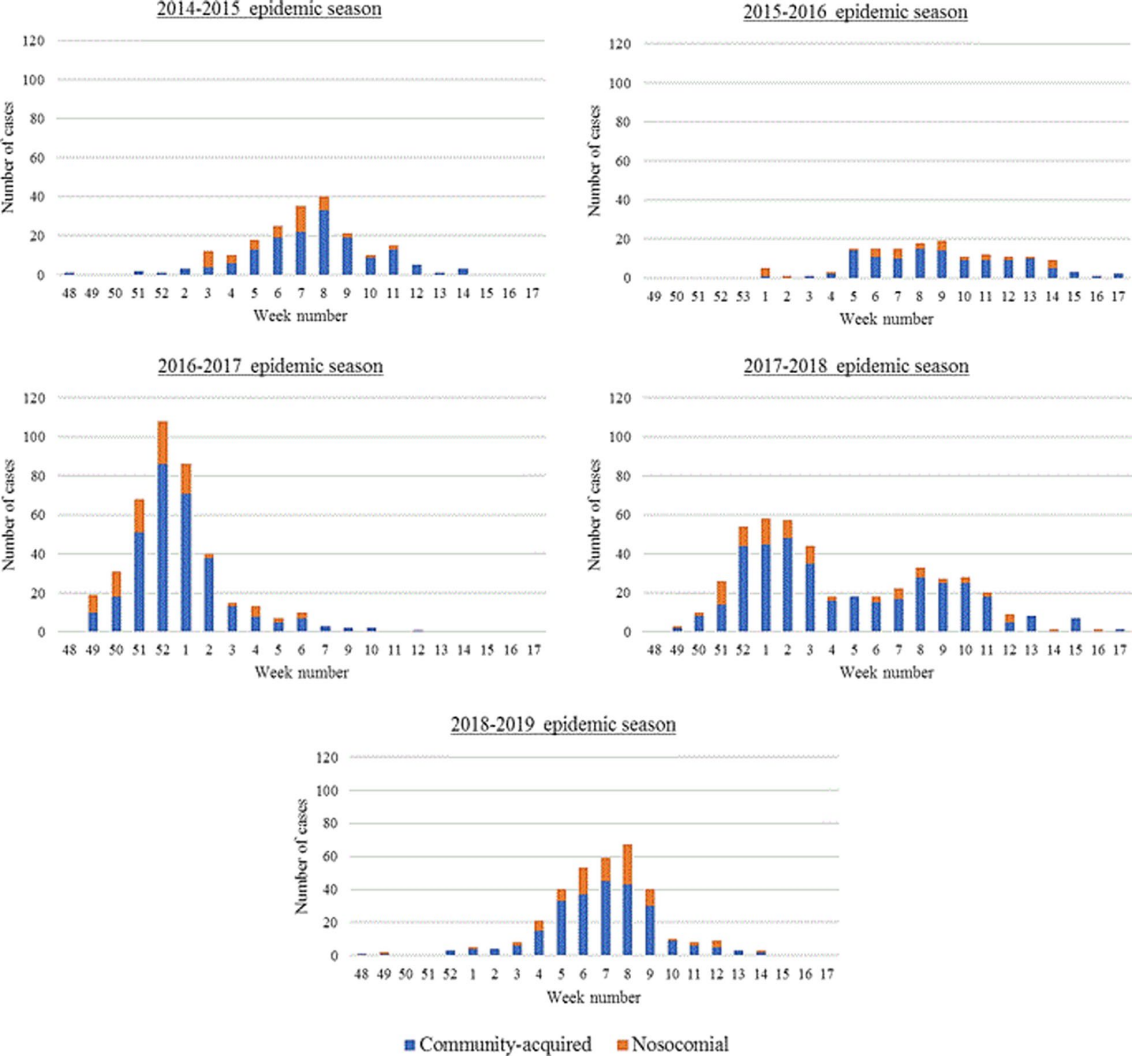
Weekly distribution of nosocomial and community-acquired influenza cases. Figure presents the weekly distribution of nosocomial and community-acquired influenza cases over the 5 epidemic seasons considered, from 2014 to 2019, at Grenoble Alpes University Hospital

A total of 1540 patients, resulting in 1559 stays, with a positive influenza test were included: 202 in 2014–2015, 152 in 2015–2016, 405 in 2016–2017, 464 in 2017–2018, 336 in 2018–2019. The weekly distribution of cases is presented in Fig. 1.

The outbreak profile as well as the number of cases were very variable over the study period as shown in Fig. 1. Cases presentation and severity (Table 2) varied across seasons with significant differences in median age (*p* = 0.0001), existence of severity risk factors (*p* < 0.0001), proportion of antiviral treatment (*p* < 0.0001) and mortality (*p* = 0.014). Type A virus was widely predominant in 2014–2015, 2016–2017 and 2018–2019; both types A and B viruses were circulating with a predominance of type B in 2015–2016 and 2017–2018. A prescription of droplet precautions was found for 66.67 to 78.57% of cases (*p* = 0.025). There was no statistically significant difference in the proportion of precautions prescriptions between risk units and others (72.25% *vs.* 70.71%, *p* = 0.533). However, mean time between diagnosis and precautions prescription was significantly lower in risk units: 0.63 days (± 0.97) *versus* 0.87 days (± 1.20) in other units (*p* = 0.0006). The proportion of rapid tests used during the different seasons (except the reference season when rapid tests were not used) was systematically higher in risk units than in non-risk units: 20.10% (121/602) *versus* 13.30% (60/451) in 2015–2016, 10.64% (95/893) *versus* 4.84% (44/909) in 2016–2017, 18.79% (318/1692) *versus* 5.89% (70/1189) in 2017–2018 and 64.63% (1215/1880) *versus* 10.13% (125/1234) in 2018–2019.

**Table 2:** Patients’ and epidemics characteristics over the 5 influenza epidemic seasons

	2014–2015 N = 202	2015–2016 N = 152	2016–2017 N = 405	2017–2018 N = 464	2018–2019 N = 336	*p* value
Age in years, median (IQR)	79.93 (56.76–87.66)	71.21 (49.96–81.01)	81.45 (67.35–87.78)	78.45 (64.43–87.49)	79.27 (65.45–86.66)	0.0001*
Male sex, N (%)	86 (42.57)	76 (50.00)	185 (45.68)	218 (46.98)	160 (47.62)	0.677**
Virus type, N (%)						
- A	180 (89.11)	72 (47.37)	405 (100.00)	141 (30.39)	336 (100.00)	
- B	17 (8.42)	80 (52.63)	0 (0.00)	323 (69.61)	0 (0.00)	–
- A/B	5 (2.48)	0 (0.00)	0 (0.00)	0 (0.00)	0 (0.00)	
Nosocomial cases, N (%)	48 (23.76)	36 (23.68)	90 (22.22)	84 (18.10)	89 (26.49)	0.074**
Severity risk factor(s), N (%)	NA	118 (78.67)	388 (95.80)	424 (91.38)	324 (96.43)	< 0.0001**
Patients vaccination, N (%)	NA	52 (41.60)	164 (53.25)	170 (48.02)	128 (53.56)	0.086**
Oseltamivir treatment, N (%)	NA	124 (84.35)	356 (88.12)	337 (73.26)	284 (84.52)	< 0.0001**
Droplet precautions, N (%)	NA	110 (78.57)	282 (70.15)	343 (74.24)	224 (66.67)	0.025**
ICU stay, N (%)	NA	18 (15.65)	49 (12.10)	42 (9.05)	35 (10.42)	0.173**
Death, N (%)	NA	4 (3.96)	37 (9.14)	33 (7.11)	12 (3.57)	0.014**

Table describes the patients’ and epidemics characteristics over the 5 influenza epidemic seasons considered from 2014 to 2019, at Grenoble Alpes University Hospital

Bold is used to highlight statistically significant results

*IQR* interquartile range, *ICU* intensive care unit

*Kruskal–Wallis test; **Khi-2 test

Several units had clusters (> 2 cases) of nosocomial influenza transmission: 3 clusters in 2015–2016 (none in risk units), 5 clusters in 2016–2017 (1 in a risk unit), 1 cluster in 2017–2018 (in a risk unit) and 8 clusters in 2018–2019 (3 in risk units). In most clusters, one or more HCWs with influenza-like illness were identified. However, their role in the transmission could not be confirmed given that influenza diagnostic tests were not routinely performed for HCWs.

### Nosocomial influenza

In bivariate analysis (Table 2), there was no significant reduction in nosocomial influenza over the 5 seasons with a proportion of nosocomial cases ranging from 18.10 to 26.49% (*p* = 0.074). Comparing proportions of nosocomial cases between risk units and others for each season showed that this proportion was systematically lower in risk units when the multimodal strategy was implemented: 15.96% (15/94) *versus* 36.21% (21/58) in 2015–2016 (*p* = 0.004), 14.56% (30/206) *versus* 30.15% (60/199) in 2016–2017 (*p* < 0.0001), 13.62% (38/279) *versus* 24.86% (46/185) in 2017–2018 (*p* = 0.002) and 18.99% (34/179) *versus* 35.03% (55/157) in 2018–2019 (*p* = 0.001). In 2014–2015, when the strategy was not implemented yet, this proportion was not significantly different between risk units and others (23.53% and 24.10%, respectively; *p* = 0.926).

Similar results were found in Poisson regression. Being hospitalized in a risk unit in 2014–2015 was not significantly associated with nosocomial influenza (IRR = 1.13, 95% CI = 0.64–2.00) as shown in Table 3. There was also no significant association between epidemic season and nosocomial influenza in non-risk units (implementing 3 measures): IRR = 1.17, 95% CI = 0.63–2.16 in 2015–2016; IRR = 0.96, 95% CI = 0.55–1.67 in 2016–2017; IRR = 1.18, 95% CI = 0.70–2.00 in 2017–2018 and IRR = 1.52, 95% CI = 0.91–2.56 in 2018–2019. In risk units (implementing 5 measures) however, there was a significant reduction in nosocomial influenza over 2 epidemic seasons compared to 2014–2015 (IRR = 0.39, 95% CI = 0.19–0.81 in 2016–2017; IRR = 0.48, 95% CI = 0.23–0.97 in 2018–2019). In 2015–2016 and 2017–2018, there was also a reduction in nosocomial influenza in risk units, but this difference was not significant (IRR = 0.56, 95% CI = 0.23–1.34 and IRR = 0.50, 95% CI = 0.24–1.03; respectively). The total number of cases was used for adjustment and this variable was associated with a higher risk of nosocomial influenza (IRR = 1.07, 95% CI = 1.06–1.07, for each supplementary case). Level of autocorrelation was moderate and considered acceptable.

**Table 3:** Number of nosocomial influenza cases analyzed by Poisson regression

	IRR	95% confidence interval	*p* value
Unit^a^			
- Non-risk unit	1	–	–
- Risk unit	1.13	0.64–2.00	0.681
Epidemic season^b^			
- 2014–2015	1	–	–
- 2015–2016	1.17	0.63–2.16	0.619
- 2016–2017	0.96	0.55–1.67	0.881
- 2017–2018	1.18	0.70–2.00	0.540
- 2018–2019	1.52	0.91–2.56	0.111
Risk unit * epidemic season^c^			
- Risk unit * 2014–2015	1	–	–
- Risk unit * 2015–2016	0.56	0.23–1.34	0.191
- Risk unit * 2016–2017	0.39	0.19–0.81	**0.011**
- Risk unit * 2017–2018	0.50	0.24–1.03	0.060
- Risk unit * 2018–2019	0.48	0.23–0.97	**0.042**
Total number of influenza cases	1.07	1.06 – 1.07	**< 0.0001**

Table presents the number of nosocomial influenza cases analyzed by Poisson regression over the 5 epidemic seasons considered from 2014 to 2019, at Grenoble Alpes University Hospital

Bold is used to highlight statistically significant results

*IRR* incidence rate ratio

^a^For the reference season 2014/2015

^b^For the non-risk units

^c^Interaction term

### HCWs’ vaccination

HCWs’ vaccination rates have increased from 18.31% in 2014–2015 to 34.70% in 2018–2019, with rates at 21.96% in 2015–2016, 22.80% in 2016–2017 and 29.10% in 2017–2018.

### Surgical mask use compliance in risk units

**Fig. 2:**
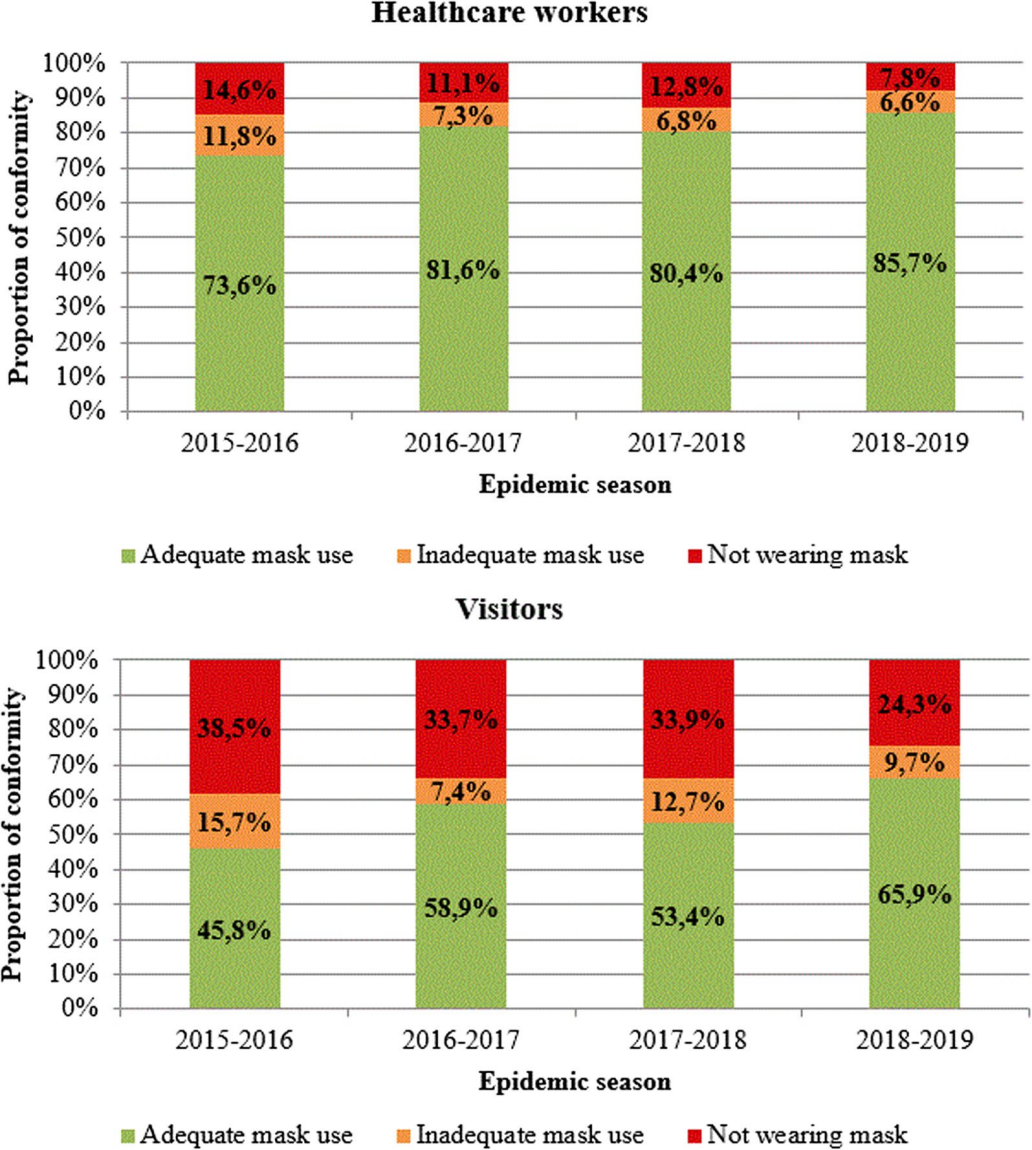
Conformity rates of surgical masks use for healthcare workers and visitors. Figure presents the conformity rates of surgical masks use for healthcare workers and visitors over the 4 influenza epidemic seasons during which the multimodal strategy was applied, from 2015 to 2019, at Grenoble Alpes University Hospital

Data on compliance with surgical mask use by HCWs and visitors are presented in Fig. 2. A total of 12,997 observations were made on HCWs (2969 in 2015–2016, 2461 in 2016–2017, 3995 in 2017–2018 and 3572 in 2018–2019) and 3791 on visitors (1145 in 2015–2016, 489 in 2016–2017, 1479 in 2017–2018 and 678 in 2018–2019). Proportion of adequate use for HCWs was high (over 80%, except for the first season of implementation) and has increased throughout the study period from 73.6% in 2015–2016 to 85.7% in 2018–2019. Rates were lower overall but adequate use for visitors has also increased from 45.8% in 2015–2016 to 65.9% in 2018–2019.

## Discussion

The strategy with 5 modalities, including systematic surgical mask use and improvement of diagnosis capacities, seemed to have an impact on the risk of nosocomial influenza. In the corresponding units, the risk of nosocomial influenza was reduced by half and this result was significant for 2 epidemic seasons. In units implementing only 3 measures however, there was no significant difference in IRRs over the study period. The absence of difference between the 2 types of units in 2014–2015, when the strategy was not implemented yet, suggests that they were initially comparable for the risk of nosocomial influenza and the differences over the other seasons can be reasonably related to the application of different strategies.

Although the strategy with 3 modalities did not seem effective to reduce the risk of nosocomial influenza, no conclusion can be drawn on its actual effectiveness given the number of variations between epidemic seasons that cannot be adjusted on. The epidemic curves presented in Fig. 1 showed variations in epidemics dynamic and we observed significant differences in patients’ age, influenza types, vaccination rates and adherence to droplet precautions. The time between diagnosis and precautions prescription was longer in units implementing 3 modalities and could explain some difference in the risk of nosocomial influenza. Of note, the institutional policy regarding droplet precautions has changed from the 2017–2018 epidemic season. Given the lack of single occupancy rooms, patients with influenza can now be hospitalized in double occupancy rooms provided that a screen is deployed between beds, the neighbour is immunocompetent and receives a prophylactic treatment. This strategy was not associated with a higher risk of nosocomial influenza in neighbours [33].

The 2 measures differentiating the units were systematic surgical mask use and improvement of diagnosis capacities. Surgical masks seemed to be well accepted by HCWs as shows the high compliance with this measure. Although there is limited evidence on the protective role of surgical masks against influenza acquisition, correct use may improve these results [16, 17]. In addition, it was demonstrated that surgical mask use was associated with reduced aerosol virus shedding [34] and a study by Ambrosch et al. found that systematic surgical mask use for HCWs was associated with a reduction in nosocomial influenza [18]. The role of rapid diagnostic tests in prevention of nosocomial influenza could be explained by a shorter time to diagnosis resulting in shorter time to adequate precautions. Several publications have underlined the importance of rapid tests in the management of influenza outbreaks [18–21] and their impact on antibiotic consumption [35, 36]. The total number of influenza tests used each season increased over the study period, as well as the number of rapid tests, particularly in 2018–2019 when point-of-care were implemented in the emergency department. The proportion of rapid tests used was systematically higher in risk units, as expected.

Substantial improvement in HCWs’ vaccination coverage, that almost doubled, was observed over the study period. The implementation of delocalized vaccination in units by designated nurses in 2017–2018 could explain the strong increase observed in this season. This is consistent with literature as a review underlined the importance of improving accessibility to vaccination [37]. Despite these results, coverage remains insufficient regarding the 60% target set by the Healthcare Infection Control Practices Advisory Committee [13]. Similar findings have been reported in Europe and raise the question of a possible mandatory policy [38].

To our knowledge, this is the first study, with over 1500 patients included, to assess a global prevention strategy against nosocomial influenza unlike many publications evaluating one or 2 measures only. Our findings suggested an impact on both HCWs’ awareness (which is reflected in the increase of influenza tests, increase of vaccination coverage and adherence towards surgical masks use) and the risk of nosocomial influenza. A large database was constituted since 2015–2016 and the continuation of the surveillance will allow an assessment of the strategy in the long run.

Our study had also some limitations. As data were not collected for research purposes, it was not homogeneous between 2014/2015 and other seasons. In 2014/2015, there was only a minimal surveillance in order to identify nosocomial cases, but the definition used to define nosocomial cases was the same so the value for the baseline rate should be comparable to the following seasons. Including data from several seasons before the implementation of the strategy would have given much more strength to our results, as we cannot exclude that the 2014/2015 season was associated with a higher risk of nosocomial influenza than the previous ones. Unfortunately, data prior to 2014/2015 were not available. We could not determine incidence rates for nosocomial influenza as the denominator (number of people susceptible to be infected) was unknown. Regarding vaccination, data were not available at unit level so we cannot exclude that coverage was not homogeneous between the 2 groups with a possible impact on the risk of nosocomial influenza, and annual vaccine effectiveness was not taken into account. As the 5 measures were implemented simultaneously, assessment of the impact of each measure was difficult. However, the existence of 2 groups allowed us to suspect the positive role of the 2 measures differentiating the groups: surgical mask use and rapid diagnostic tests. We cannot exclude that only these 2 measures had a real impact on the risk of nosocomial influenza, but a possible interaction between the different measures must be kept in mind. Indeed, units implementing 5 measures are units which admit more often patients with influenza, so they are likely more aware about the risk of nosocomial influenza, and they might therefore have a better vaccination coverage or a better compliance with the prevention measures. The results of this study do not exclude a positive impact of HCWs vaccination, communication and surveillance, but advocate for a better assessment of the effect of each measure. Lastly, the possible extrapolation of our findings is unknown as our study was monocentric.

## Conclusions

Our data suggest that the application of a strategy with 5 modalities, including promotion of HCWs’ vaccination, epidemiologic surveillance, communication, systematic surgical mask use and rapid diagnosis, could have an impact on the risk of nosocomial influenza. However, these results need to be confirmed in a prospective way to limit bias and to better explore the role of each measure. Generalizing such strategy would also generate additional costs with increased consumption of tests and masks as well as workload for HCWs. Other strategies, such as surgical mask for non-vaccinated HCWs only, have been proposed [39] but implementation seem complex and the insight provided by the COVID-19 pandemic seems to advocate for a combination of both measures rather than an opposition.

## Data Availability

The datasets generated and/or analyzed during the current study are not publicly available due to confidentiality reasons.

## Abbreviations

CI: Confidence interval
ED: Emergency department
HCW: Healthcare workers
ICU: Intensive care unit
IQR: Interquartile range
IRR: Incidence rate ratio
NA: Not applicable
